# New proteoliposome vaccine formulation from *N. meningitidis* serogroup B, without aluminum hydroxide, retains its antimeningococcal protectogenic potential as well as Th-1 adjuvant capacity

**DOI:** 10.1186/1471-2172-14-S1-S12

**Published:** 2013-02-25

**Authors:** Beatriz Tamargo, Yanet Márquez, Wendy Ramírez, Bárbara Cedré, Manuel Fresno, Gustavo Sierra

**Affiliations:** 1 Institute for Pharmacy and Foods, University of Havana, Cuba; 2 National Center for Biologicals, Mayabeque, Cuba; 3 Center for Molecular Biology “Severo Ochoa”, Madrid, Spain; 4 Finlay Institute, Havana, Cuba

## Abstract

Proteoliposomes purified from the Outer Membrane of *Neisseria meningitidis* B, have been successfully used as core for adjuvants and vaccine formulations. We have tried to increase their structural definition and to conserve their efficacy and stability avoiding the addition of the aluminum hydroxide to the final formulation. Liposomal particle systems were prepared from components of defined molecular structure, such as a *Neisseria meningitidis* B protein complex, extracted and purified without forming vesicle structures. Liposomes were prepared from a mixture of dioleoyl phosphatidyl serine and cholesterol, using the classical dehydration-rehydration method. Transmission Electron Microscopy (TEM) was used to characterize the liposomes. BALB/c mice were used for animal testing procedures. Analysis of specific IgG response, serum bactericidal activity as well as DTH reaction was carried out. Isolation and purification of mRNA and real-time PCR, was performed to determine the dominating Th lymphokine pattern. The new antimeningococcal formulation without aluminum hydroxide prepared with components of defined molecular structure assembled itself into Neoproteoliposomes (NPL) ranging from 50 to 70 nm in diameter. The extraction and purification of selected membrane proteins to provide the antigen for this new formulation (PD-Tp), as well as the NPL-formulation favors a Th1 response pattern, suggested by the higher percentages of DTH, increased expression of proinflamatory lymphokine mRNAs when administered by intramuscular and intranasal routes. It stimulates a systemic bactericidal antibody response against *Neisseria meningitidis* B and immunologic memory similar to the Cuban VA-MENGOC-BC^®^ vaccine, even at lower dosages and is less reactogenic at the injection site in comparison with the formulation with aluminum hydroxide. This new adjuvant formulation could be applicable to the development of new and improved vaccines against meningococcal disease, and eventually as modulators of the immune response against other diseases.

## Introduction

Production of serogroup B *N. meningitidis* membrane proteins into vesicles or proteoliposomes (PL) and their use as the main antigenic component in the Cuban meningococcal vaccine VA-MENGOC-BC^®^, has been one of the most efficient approaches to protect against disease produced by this serogroup [1,2]. The capacity of PL to activate a Th1 immune response has been characterized and includes the production of gamma interferon (γIFN), induction of delayed hypersensitivity (DTH), opsonophagocytosis and serum bactericidal activity; a mechanism generally accepted as protective, mediated by IgG2a in mice and IgG1 and IgG3 in humans [3,4]. This vaccine is adjuvanted twice: by the proteoliposomal conformation adopted by membrane proteins and by aluminum hydroxide gel. At present, different adjuvants, based on nanoparticle systems, have been developed to release antigens in subunit vaccines. Among them liposomes, proteoliposomes and other delivery systems have great acceptance, since they work as non-living antigen vectors [5]. However, it is necessary to increase molecular definition preserving efficiency and stability, without adding aluminum hydroxide to the final formulation, thus reducing reactogenicity [6]. The aim of the present study was to investigate the immune response and local reactogenicity induced in BALB/c mice, immunized with an adjuvant formulation prepared from components of the *N. meningitides* B with defined molecular structure, which are assembled in the form of proteoliposomes, without adding aluminum hydroxide gel.

## Material and methods

Liposomal particle systems were prepared from a *Neisseria meningitidis* B protein complex, extracted and purified without forming vesicle structures (PD) and assembled in the form of proteoliposomal nanoparticles designated as NPL. A buffer suspension of the protein complex (Tris-HCL 5mM, detergent 0.1%, pH 7.4), named PD-Tp and in addition the Cuban meningococcal VA-MENGOC-BC^®^ vaccine, and the OMV (Outer Membrane Vesicles or proteoliposomes) supplied by Finlay Institute, Cuba, were used as controls. Neoproteoliposomes (NPL) were prepared from a mixture of dioleoyl phosphatidyl serine (DOPS) and cholesterol (Sigma Aldrich Co.UK), using the dehydration-rehydration procedure described by Kirby and Gregoriadis in 1984 [7]. The particulate shape and size was established by transmission electron microscopy (TEM) and negative staining was performed using the classical drop method with 2% (v/v) phosphotungstic acid.

**Figure 1:**
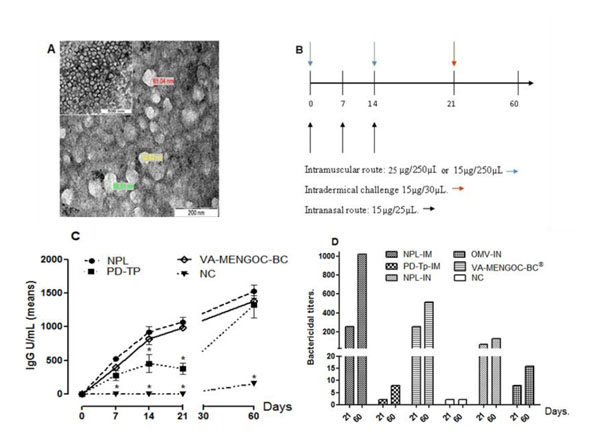
**Antimeningococcal protectogenic potential and adjuvant capacity of the new proteoliposomic (NPL) formulation from *N. meningitidis* serogroup B, without aluminum hydroxide. A:** Micrograph obtained by TEM, showing abundant spherical particles of a size ranging between 50 and 70 nm of diameter. **B** Diagram showing the scheme of immunization designed for the evaluation of the formulations in BALB/c mice. In **C**, the graph represents the mean and SD of the level of specific antibodies expressed in units of IgG anti OMV in the serum of the immunized mice at 7, 14, 21 and 60 days, after the last dose of immunization, (*) indicate significant differences for p< 0.05. The results of the determination of the bactericidal activity of serum are shown in **D,** each bar represents the mean of three individual experiments, accomplished for each group of assay, at the 21 days after the last immunization dose and at the 60 days after receiving the antigenic intradermical challenge for DTH assay. **NPL:** Mice inoculated with the formulation of neo-proteoliposomes, **PD-Tp:** Mice immunized with the protein complex of the *N. meningitidis* serogroup B (PD) in buffer (Tris HCL 5mM, 0.1% detergent, pH 7.4), **VA-MENGOC-BC^®^:** Mice inoculated with the Cuban antimeningococcal BC vaccine, **OMV:** Mice inoculated with Outer Membrane Vesicles of the *N. meningitidis* serogroup B, **NC:** Control group that received buffer solution. **IM:** Intramuscular administration route, **IN:** Intranasal administration route.

BALB/c mice (10 per group) were distributed in three immunization groups and a control. The first, second and third group were immunized with two intramuscular (IM) doses of NPL, PD-Tp (15 µg in 250µl) respectively and VA-MENGOC-BC^®^, (25 µg in 250µl) with a 14 day interval. The fourth group was immunized with a buffer solution and was used as a negative control identified as NC. Two additional groups were immunized with three intranasal (IN) doses of NPL or OMV (15µg in 25µl, 12.5µl in each nostril) with a 7 day interval. The serum samples were collected 7, 14, 21 and 60 days after the last immunization. (Fig.1B) Specific antimeningococcal-OMV-IgG antibody titers in sera from immunized mice were determined by a capture enzyme-linked immunosorbent assay (ELISA), according to the Finlay Institute protocol. Calculations were performed using the CDC ELISA Data Processing and Analysis Program, Version 2.15-09/27/2004 [8,9]. The serum bactericidal antibody (SBA) assay was carried out as previously reported [10], but using a Cu385-83 serogroup B *N. meningitidis* strain suspension containing 2x10^8^CFU/mL in phosphate buffered saline (PBS) supplemented with a rabbit complement (Pel Freez) (with no bactericidal activity in itself). The bactericidal antibody titer was calculated as the reciprocal of the highest dilution of serum causing 50% or more colony death by bacteriolysis compared with counting of CFU in relation to the control sample**.** The results were expressed as 1/Titer. Twenty one days after the last dose the DTH reaction was induced and measured at 48 h after intradermical challenge with PD, in immunized mice; the footpad swelling test was carried out as previously described [11]. The determination of cytokine expression was performed by RT-PCR. Briefly, the spleen was removed from five animals in each group and the splenocytes purified. These were cultured in RPMI medium and stimulated with 12μg of the antigen (PD). Total RNA was extracted from the spleen cells in 1 ml of TRIzol^®^ reagent (Invitrogen). Analysis of mRNA expression was carried out using TaqMan Gene Expression Assays (Applied Biosystems, Ca., USA) as described previously [12]. In this method, mRNA levels for each sample were normalized to glyceraldehyde-3-phosphate dehydrogenase (GAPDH) levels and then expressed as a relative increase or decrease compared with the negative control levels. Each experiment was repeated a minimum of three times. For *in vivo* experiments, error bars represent the mean, plus the SD of the readings from a minimum of five mice. The significance of the difference between the means of the control group and the experimental groups or between two experimental groups is indicated with an asterisk and was calculated by the non-parametric Mann-Whitney test. In all cases, a p< 0.05 significance level was used.

## Results and discussion

NPL obtained in this work showed homogeneity with respect to shape and size, ranging from 50 to 70 nm of mean diameter, making them appropriate for antigen presentation to the immune system (Fig. 1A). Intramuscular administration of the NPL formulation was capable of inducing specific antimeningococcal-OMV-IgG antibody levels in immunized animals, at the same level as VA-MENGOC-BC^®^; its concentration was higher and different from those of the NC group, and from the non adjuvanted protein PD at 7, 14, 21 days after immunization. The subcutaneous footpad inoculation with PD at 21 days, challenging for the DTH assay, resulted in a very effective booster dose as demonstrated by the antibody level increase measured at 60 days. Antibody levels in animals immunized with NPL, VA-MENGOC-BC^®^ and PD were not significantly different with each other, but only with those of the NC group.

These findings confirm the NPL and VA-MENGOC-BC^®^ capacity for inducing immunological memory, since all the animals received an intradermal PD booster for the DTH assay. This reaction allowed *in vivo* confirmation of Th1 clone functioning after activation by their capacity to generate inflammatory processes when faced with a new antigenic challenge. There did not appear to be any immediate hypersensitivity reaction, proving that the inflammatory processes were provoked after 48 hours. Both NPL and PD-Tp induced higher responses than VA-MENGOC-BC®, and NC (Fig.1C).

SBA assays using pooled mouse sera from the 21 and 60 day time points after the last dose showed that those animals immunized with either NPL or VA-MENGOC-BC^®^, had the greatest bactericidal activity against the Cu385-83 epidemic strain. The sera from the groups immunized with the NPL and VA-MENGOC-BC^®^ IM, (titres of 256 ± SD ≤ 0.5 log_2_ at 21 days and titre of 512 ± SD ≤ 0.5 log_2_ VA-MENGOC-BC^®^ IM, and titre of 1024 ± SD ≤ 0.5 log_2_ NPL at 60 days)**,** the PD-Tp sera at 21 days did not elicit bactericidal titres (SBT), while the bactericidal titre at 60 days was 8 ± SD ≤ 0.5log_2_**.** The IN route of immunization was less effective in inducing SBA titer sat this dosage since NPL only elicited titres of 64 ± SD ≤ 0.5log_2_ at 21 days and 128 ± SD ≤ 0.5log_2_ at 60 days while the OMV induced titres of 8 ± SD ≤ 0.5log_2_ at 21 days and 16 ± SD ≤ 0.5 log_2_ at 60 days (Fig.1D).

The SBA is a correlate for protective antibody responses against *Neisseria meningitides.* SBA titers above 8 or a 4 fold increase are considered as protective in many species [10]. We found that IM immunization with NPL and VA-MENGOC-BC^®^ yielded sera with greater bactericidal activity than the IN immunized groups with the same formulations. The observed differences between SBA for the different immunization routes IM vs. IN suggest that composition differences exist among antibody production, which motivated us to perform, in a separate study, an IgG subclass analysis. We found that those animals immunized IM with the NPL and VA-MENGOC-BC^®^ elicited higher levels of IgG2a than did those animals immunized IN at 15 micrograms per dose (results not shown). In mice, the IgG2a isotype is the most efficient activator of complement, while the IgG1 isotype is the poorest. Thus, the lower IgG2a detected in IN treatment could in part explain the inferior bactericidal activity observed in these sera. Similar results were obtained by del Campo and coworkers [13] immunizing with OMV and its derivative cochleates at 50µg dose by the IN route.

Th1/Th2 patterns coexist in the immune response, but one predominates over the other, depending on the antigenic stimulation of the pathogen responsible for the disease and, in the case of vaccines, on the formulation used. The three assay formulations evaluated in this study stimulate IL-10, TGFβ, IFN-γ and TNF expression. These last pro-inflammatory cytokines, related to cellular response, significantly increase their expression in the groups vaccinated with PD-Tp and NPL, when they are administered by the IM, as well as IN route. Meanwhile the relative amounts of IL-10 and TGFβ messengers are reduced or maintained relatively equal to with respect to VA-MENGOC-BC^®^. These last cytokines favor more regulatory pressure on the immune response pattern.

In relation to these results, the new antimeningococcal formulation (NPL) without aluminum hydroxide prepared with components of defined molecular structure, and assembled in the form of nanoproteoliposomes, are equal in terms of immunogenicity to the classic formulation that uses aluminum hydroxide, even at a lower dosage, giving a more potent T-cell immune response with equivalent immunological memory, lower reactogenicity and better structural definition. This new formulation could be applicable to new vaccines against meningococcal disease, and eventually as modulators of the immune response against other diseases.

## Authors' contributions

BT and GS conceived of the study, and participated equally in its design, carried out pharmaceutical formulations and immunological analyses and drafted the manuscript.BT participated actively in all studies and analysis.GS coordinated the strategy; YM and WR participated in immunological studies; BC carried out serum bactericidal studies; MF participated in design of study of the lymphokine pattern and helped in its realization and analysis.

All authors have read and approved the final manuscript.

## Competing interests

The authors declare that they have no competing financial interests.

## Declarations

This article has been published as part of *BMC Immunology* Volume 14 Supplement 1, 2013: Proceedings from Delivery Systems and Current strategies to drug design. The full contents of the supplement are available online at http://www.biomedcentral.com/bmcimmunol/supplements/14/S1.

## References

[B1] Sierra GV Campa HC Valcárcel NM García IL Izquierdo PL Sotolongo PF Casanueva GV Rico CO Rodriguez CR Terry MH Vaccines against group B *Neisseria meningitidis*: protection trial and mass vaccination results in Cuba NIPH Ann 1991 14 195 210 1812432

[B2] Tamargo B Sierra VG Group B Meningococcal vaccine based on proteoliposomes The New England Journal of Medicine 2007 357 17 1779 1780 17960023 10.1056/NEJMc072063

[B3] Rodríguez T Perez O Ugrinovic S Bracho G Mastroeni P Bacterial derived Proteoliposomes as ideal delivery system and cellular adjuvant Vaccine 2006 24 2 24 25 10.1016/j.vaccine.2005.01.106 16823912

[B4] Pérez O Lastre M Lapinet J Bracho G Díaz M Zayas C Taboada C Sierra G Immune response induction and new effector mechanisms possibly involved in protection conferred by the Cuban anti-meningococcal BC vaccine Infect Immun 2001 69 4502 8 10.1128/IAI.69.7.4502-4508.2001 11401992 PMC98525

[B5] Redes T Hook S Lipid nanoparticles as delivery system for subunit vaccines VacciMonitor 2009 18 1 38

[B6] Batista A Lindblad EB Oviedo E Progress in understanding adjuvant immunotoxicity mechanisms Toxicology Letters 2011 203 97 105 10.1016/j.toxlet.2011.03.001 21392560

[B7] Kirby C Gregoriadis G Dehydration-rehydration vesicles (DRV): A new method for high yield drug entrapment in liposomes Biotechnology 1984 2 979 84 10.1038/nbt1184-979

[B8] Ferriol X García AG Ochoa R Bravo I Blanco R Estrada E Validación de un ELISA para la cuantificación de IgG humana anti proteína de Neisseria meningitidis serogrupo B Rev Cub Med Trop 1999 51 99 105 10887568

[B9] Nerey M Ochoa R Martínez JC Licea T Ferriol X García AM Validación de un ELISA para la cuantificación de IgG humana anti polisacárido capsular de *Neisseria meningitidis* serogrupo C Biotecnol. Apl 1999 16 113 5

[B10] Borrow R Carlone GM Rosenstein N Blake M Feavers I Martin D Zollinger W Robbins J Aaberge I Granoff DM *Neisseria meningitidis* group B correlates of protection and assay standardization—International Meeting Report Emory University, Atlanta, Georgia, United States, 16–17 March 2005 Vaccine 2006 24 5093 5107 10.1016/j.vaccine.2006.03.091 16838413

[B11] Lincopan N Santana M Faquím-Mauro E B da Costa MH Carmona-Ribeiro AM Silica-based cationic bilayers as immunoadjuvants BMC Biotechnology 2009 5 2 19 10.1186/1472-6750-9-5 PMC2647919 19152701

[B12] van den Broeck W Derore A Simoens P Anatomy and nomenclature of murine lymph nodes: Descriptive nomenclatory standardization in Balb/c AnNCrl mice J Immuno. Methods 2006 312 1-2 12 19 10.1016/j.jim.2006.01.022 16624319

[B13] del Campo J Zayas C Romeu B Acevedo R González E Bracho G Cuello M Cabrera O Balboa J Lastre M Mucosal immunization using proteoliposome and cochleate structures from Neisseria meningitidis serogroup B induce mucosal and systemic responses Methods 2009 49 301 308 10.1016/j.ymeth.2009.03.025 19410000

